# Optimization Conditions of Ultrasound-Assisted Extraction for Phenolic Compounds and Antioxidant Activity from *Rubus alceifolius* Poir Leaves

**DOI:** 10.1155/2023/7576179

**Published:** 2023-10-10

**Authors:** Chi Hai Tran, Minh Tri Nghiem, Anh Minh Trinh Dinh, Thi Thuy Nga Dang, Thi Thuy Van Do, Thi Nga Chu, Tien Hung Mai, Van Man Phan

**Affiliations:** ^1^Faculty of Food Science and Technology, Ho Chi Minh City University of Industry and Trade, 700000, Vietnam; ^2^Faculty of Food Technology, Ba Ria–Vung Tau College of Technology, 790000, Vietnam

## Abstract

*Rubus alceifolius* Poir (*R.A.* Poir) leaves are rich in phenolic compounds, offering many health benefits due to their incredible antioxidant potential. In this study, conditions for the ultrasound-assisted extraction (UAE) of phenolic compounds and antioxidant activity from *R.A.* Poir leaves were optimized using response surface methodology (RSM). This methodology assessed the effects of ultrasound power (*X*_1_: 100-500 W), extraction temperature (*X*_2_: 30-60°C), and extraction time (*X*_3_: 5-55 min). The optimized UAE conditions were then compared with conventional extraction methods (Soxhlet extraction: SE and maceration extraction: ME) for extracting total phenolics. A phenolic profile using GC-MS and antioxidant activity (ABTS) was also compared. According to the RSM, the best conditions for UAE to extract the highest total polyphenol content and ABTS radical scavenging activity were 320 W ultrasound power, 40°C extraction temperature, and 35.5 min sonication duration. Under these optimal conditions, the TPC and antioxidant activity reached 16.68 mg GAE/g dm and 21.9 mg TE/g, respectively, closely aligning with the predicted values. The UAE extraction technique proved to be more efficient in extracting phenolics and antioxidant capacity (ABTS (2,2′-azino-bis(3-ethylbenzthiazoline-6-sulphonic acid)) radical scavenging activity, and enzyme inhibition) compared to the conventional extraction methods (SE and ME). A GC-MS analysis identified 12 components, including 5 phenolics and 3 flavonoids, which likely contribute to the antioxidant activity. Consequently, the UAE method improved extraction efficiency within a shorter time frame, suggesting that UAE is a promising, efficient, and ecofriendly technology for extracting bioactive compounds from *R.A.* Poir leaves.

## 1. Introduction

*Rubus alceifolius* Poir (*R.A.* Poir) belongs to the Rubus L. (Rosaceae) family and is widely distributed in China, Vietnam, and Malaysia [1]. *Rubus alceifolius* prefers to grow in humid and shady environments. The fruit of the *R.A.* Poir is a red or purple drupe, similar in appearance to a raspberry or blackberry, and has a sweet and tangy flavor [1, 2]. These fruits are celebrated for their high vitamin C and phenolic compound content [1–3]. Meanwhile, the main components of the leaves of *R.A.* Poir are phenolics, flavonoids, triterpenes, and others [2, 3]. Among them, phenolic compounds exhibit strong antioxidant properties and antimicrobial activity [2, 4]. Recent studies have demonstrated the leaf extracts are employed in treating ailments such as mucosal inflammation, oral lichen planus [5], atherosclerosis, and hypertension diseases [6]. Additionally, these extracts have been used to reduce the risk of fatty liver disease and other chronic [7]. Therefore, these leaves of plants attracted considerable attention as a source of phenolics.

Soxhlet and maceration are the most common methods used for phenolic extraction from plants [8, 9]. However, the disadvantages of these techniques are time-consuming and have low extraction efficiency [10, 11]. Therefore, several new techniques such as ultrasound-assisted extraction [9], supercritical fluid extraction [12], and microwave-assisted extraction [13] have been developed for phenolic extraction. Although supercritical fluid extraction and microwave-assisted extraction have high extraction efficiency, they are difficult to scale up and require expensive equipment [14, 15]. In contrast, ultrasound-assisted extraction has garnered interest due to its simplicity, low equipment cost, and ecofriendliness. Compared to traditional methods, this method uses less solvent and energy while preserving bioactive compounds' activity [14–16]. Ultrasound-assisted extraction has successfully extracted bioactive compounds from grapes [9], *Celastrus hindsii* [16], *and* date palm [17]. However, the application of ultrasound-assisted extraction for the recovery of phenolic compounds from *R.A.* Poir leaves is still limited in our current understanding.

Several variables affect the UAE process efficiency, such as ultrasound power, extraction time, and extraction temperature [16–18]. It is therefore critical to optimize these process variables to achieve maximum yields of bioactive compounds from raw materials. In recent years, response surface methodology (RSM) has been recognized as an effective tool for optimizing the extraction process [9, 16, 17]. It reduces the number of experimental trials and recognizes the influence of process parameters on the efficiency of extraction [9, 16]. The response surface methodology also serves as a visual aid to indicate the optimization region [17]. Therefore, the objectives of this study were (i) to optimize the ultrasound-assisted extraction conditions for maximum recovery of phenolics and antioxidant properties from *R.A.* Poir leaves; and (ii) to compare the extraction efficiency of the ultrasound-assisted extraction with the soxhlet and maceration methods.

## 2. Materials and Methods

### 2.1. Materials

#### 2.1.1. Plant Material

*Rubus alceifolius* Poir (*R.A.* Poir) leaves were collected from Song Hinh District, Phu Yen Province. Fresh leaves were prewashed in deionized water and then dried in a Binder ED56 oven (Germany) at 40°C for 10 hours to achieve 5–7% of moisture content. Following this, the dried leaves were ground to a fine powder, which was then sifted through a steel mesh sieve with a pore size of 1 mm. The samples were stored at 4°C until used for further analysis.

#### 2.1.2. Chemicals

Trolox (97%), ethanol (99.5%), vanillin (99%), acid acetic (99.5%), gallic acid (97%), and perchloric acid (99.99%) were procured from Anpha Chemika, India. Other reagents, such as 2,2′-azino-bis(3-ethylbenzthiazoline-6-sulphonic acid) (ABTS), and Folin–Ciocalteu, were from Sigma (St. Louis, MO, U.S.A.). Additionally, *α*-amylase solution (ex-porcine pancreas, EC 3.2.1.1) and collagenase were purchased from Sigma. All the solvents and reagents used in this study were of analytical grade.

#### 2.1.3. Ultrasound-Assisted Extraction

In this study, ethanol was used for phenolic extraction from *R.A. Poir* leaves. The ultrasonic treatment was carried out using an ultrasonic generator (VC505, Sonics & Materials Inc., Newtown, USA) at a constant frequency of 20 kHz. In each run, approximately 10 grams of sample was mixed with 200 mL of aqueous ethanol (70% ethanol concentration, 1 : 20 solid/liquid ratio), based on the optimal results of preliminary experiments. The mixtures underwent sonication at various ultrasonic powers (100, 200, 300, 400, and 500 W), temperatures (30, 35, 40, 45, 50, 55, and 60°C), and durations (5, 15, 25, 35, 45, and 55 minutes). After that, the samples were centrifuged at 3000 rpm for 5 min and then filtered using a vacuum pump. The extracts were used to determine total polyphenol content and antioxidant activity (ABTS radical scavenging activity) by colorimetric spectroscopy.

#### 2.1.4. Response Surface Methodology Analysis

The Box–Behnken design was employed to evaluate the extraction parameters on the recovery of phenolic compounds with high antioxidant activity from *R.A.* Poir leaf powder. The design consisted of 17 experiments, and each parameter was varied at three levels (low, moderate, and high) coded as -1, 0, and +1 (Table 1). The independent variables were ultrasound power (*X*_1_: 100-500 W), temperature (*X*_2_: 30-60°C), and sonication time (*X*_3_: 5-55 min). The total phenolic content (*Y*_1_) and antioxidant activity (ABTS; Y_2_) were chosen as the responses. A second-order polynomial was used to calculate the predicted response

(1) $$\begin{array}{c} Y=\beta_{0}+\overset{4}{\underset{i-1}{\sum }}\beta_{i}X_{i}+\overset{4}{\underset{i=1}{\sum }}\beta_{i}X_{i}^{2}+\overset{3}{\underset{i=1}{\sum }}\overset{4}{\underset{j=i+1}{\sum }}\beta_{ij}X^{2}, \end{array}$$

where *Y*_1_ and *Y*_2_ represent the predicted responses (total phenolic and ABTS). *β*_0_, *β*_*i*_, *β*_ii_, and *β*_ij_ are the regression coefficients. *X*_*i*_ and *X*_*j*_ function as distinct independent variables (*i* ≠ *j*). A fitted second-order polynomial was used to generate 3D surface plots, illustrating the correlation between independent variables and the subsequent response [4].

#### 2.1.5. Conventional Extraction

For soxhlet extraction (SE), 150 g *R.A.* Poir leaf powder was extracted with 400 mL of 70% ethanol for 16 hours [5]. In the case of ethanol maceration (ME), 10 g of leaf powder was extracted with 100 mL of 70% ethanol for 24 hours. The mixture was vigorously shaken in a water bath set to 200 rpm at a temperature of 30 ± 0.5°C [18]. Subsequently, the solution was filtered through a Whatman No. 1 filter paper, and the solvent was removed using a rotary evaporator (Buchi R210, Flawil, Switzerland). The extracts were used for further analysis. The experiments were conducted in triplicate.

### 2.2. Experimental Methods

#### 2.2.1. Determination of Total Phenolic Content (TPC)

The total polyphenol content was determined to the previous study described by Ibrahim et al. with slight modifications [19]. A 40 *μ*L of the extract was diluted with 1560 *μ*L of water and then mixed with 100 *μ*L of Folin-Ciocalteu reagent. After that, 300 *μ*L of 10% (*w*/*v*) sodium carbonate was added and incubated in the dark for 2 h. The solution's optical density was measured at a wavelength of 765 nm. To quantify the total polyphenol content, a standard curve was established using gallic acid (GAE) at concentrations ranging from 0.004 to 0.5 mM. This curve was used to determine the TPC of the sample, which is expressed as milligrams of gallic acid equivalents per gram of dry matter (mgGAE/g dm).

#### 2.2.2. Determination of ABTS Radical Scavenging Activity

The ABTS radical scavenging activity was determined based on the method of Pham et al. with minor modifications [20]. Briefly, a 35 *μ*L extract was mixed with ABTS radical (265 *μ*L at 7 mM) in the screw cap test tube. After that, the tubes were shaken in darkness for 10 min and measured at 734 nm using a microplate reader (PR2100, Bio-Rad, USA). For ABTS analysis, Trolox was used as the standard solution (0.1–1.0 g/100 mL in methanol). The ABTS is expressed as mg of Trolox equivalents per g of dry matter (mg/g d.m.).

#### 2.2.3. Collagenase Inhibition Assay

The anticollagenase activity was evaluated using azo dye-impregnated collagen as a substrate based on a previous study by Wang et al. [21]. Briefly, 1 mg of azo dye-impregnated collagen, 800 *μ*L of Tris-HCl (0.1 M, pH 7), and 100 *μ*L of the sample were weighed into tubes. The mixture was then shaken for 1 min before 100 *μ*L of collagenase (200 units/mL) was added. The mixture was incubated at 43°C for 1 h. The samples were centrifuged at 3000 rpm for 10 min to remove the solid. The supernatant liquid was placed on a 96-well plate, and the absorbance was measured at 550 nm. A blank was prepared by adding all reaction reagents without a sample solution.

#### 2.2.4. *α*-Amylase Inhibition Assay

*α*-Amylase inhibitory activity was determined according to the method described by Savran et al. [22] with some modifications. In brief, 25 *μ*L of plant extract was combined with 50 *μ*L of 0.05% starch solution and 50 *μ*L of *α*-amylase solution from porcine pancreas (EC 3.2.1.1, Sigma) in phosphate buffer (pH 6.9 with 6 mM sodium chloride). The reaction mixture was incubated at 37°C for 10 minutes. To complete the reaction, 25 *μ*L of 1 M HCl and 100 *μ*L of iodine-potassium iodide solution were added. A blank was also prepared by including the sample solution in all reaction reagents but excluding the *α*-amylase solution. Absorbance was measured at 630 nm using a microplate reader (PR2100, Bio-Rad, USA). The *α*-amylase inhibitory activity was reported as millimoles of acarbose equivalents (mmol ACE/g extract).

#### 2.2.5. Determination of some Chemical Components of Extracts by GC–MS Method

GC-MS analysis was conducted following the previous method with slight modifications [23]. The extracts were dissolved in ethanol and injected into an Agilent 7890A GC system equipped with an MS (Agilent Technologies). The separation of the samples was conducted on a DB-5MS column (30 m length × 0.25 mm diameter × 0.25 *μ*m film thickness). The GC-MS operating conditions were as follows: oven temperature increased from 50°C to 280°C at a rate of 10°C/min and then held isothermally for 10 min. The sample was injected in the splitless mode with 2 *μ*L, and helium, a carrier gas was at 1 mL/min. The mass spectrometer was operated at 70 eV, and the total running time of the GC was 50 min. The compounds identified by GC-MS analysis were compared with compounds in the NIST 17 mass spectral library [23]. After that, calibration curves for catechol, 2,3-dihydro-benzofuran, gallic acid, p-hydroxybenzoic acid, gentisic acid, gallic acid, vanillic acid, benzaldehyde, 2-hydroxy-4-methyl, catechin, gallocatechin, coumaroylquinic acid, and quinic acid were established to calculate the concentration of these compounds.

### 2.3. Scanning Electron Morphology (SEM)

To evaluate the morphological changes of *R.A.* Poir leaf powder, scanning electron microscopy (SEM) (JEOL, JSM 6010 LV, Technology Development Ltd., Japan) analysis was carried out. The dried samples were coated with a thin gold film using a sputter coater and then observed in a scanning electron microscope under an accelerating potential of 30 kV.

### 2.4. Statistical Analysis

In this study, each experiment was repeated 3 times, the results are presented as mean ± standard deviation. Data were analyzed *using* one-way ANOVA followed by *Duncan's* multiple range *test*. All statistical analyses were processed using Design-Expert software (version 11.0; Stat-Ease Inc., Minneapolis, MN).

## 3. Results and Discussion

### 3.1. Effect of Ultrasound Power, Temperature, and Extraction Time on Phenolic Extraction and Antioxidant Activity

**Figure 1:**
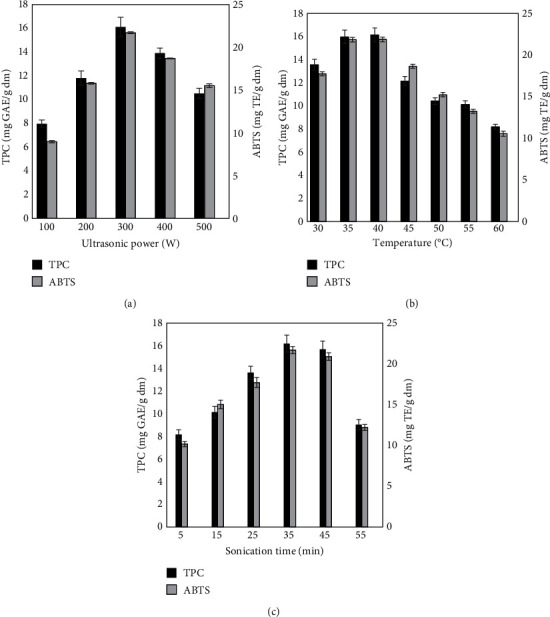
Total polyphenol content (TPC) (mg GAE/g powder) and ABTS of rubus alceifolius Poir extracted with 70% ethanol at (a) given extraction temperature (45°C) and extraction time (35 min); (b) given ultrasonic power (300 W) and extraction time (35 min); and (c) given ultrasonic power (300 W) and extraction temperature (45°C).

Figure 1(a) illustrates the effect of the ultrasound power (100-500 W) on the recovery of phenolic compounds and their antioxidant activity (ABTS) from *R.A.* Poir leaves. As depicted in Figure 1(a), there is a notable increase in TPC (total phenolic content) and ABTS values from the extracts when the ultrasonic power ranges from 100 to 300 W. The highest TPC (16.11 mg GAE/g) and ABTS (21.71 mgTE/g) are achieved at an ultrasonic power of 300 W, which is approximately twice as high as the extracted sample at 100 W. An increase in ultrasonic power could improve the mass transfer, thus facilitating the release of bioactive compounds from the extracted materials [9, 17]. However, when the ultrasonic power escalates to 500 W, the TPC and ABTS significantly decreased (*p* < 0.05). This decline may be attributed to the high ultrasonic power causing phenolic deterioration, consequently reducing the TPC and ABTS values of *R.A.* Poir leaf extract [20, 23]. Based on the obtained results, the most suitable ultrasonic power to conduct the extraction process is 300 W.

Figure 1(b) demonstrates the impact of extraction temperature on both TPC and ABTS. The TPC and ABTS increased as the temperature increased from 30 to 40°C. The peak levels for TPC and ABTS occur at 40°C, measuring 16.14 g GAE/g and 21.89 mg TE/g, respectively. However, when the extraction temperature climbs from 40 to 60°C, TPC and ABTS decreased by a factor of 1.5. This phenomenon indicated that an increase in temperature (over 40°C) led to the degradation of some heat-labile components [20, 24]. In addition, elevating the extraction temperature further hastens the evaporation solvent, which reduces the diffusivity of the solutes to be extracted. Thus, the extraction temperature of 35°C was chosen for the subsequent extraction of phenolic compounds from *R.A.* Poir leaves.

Another important factor affecting TPC and ABTS is extraction time. In this study, the samples were ultrasonically treated at 300 W at different times (5, 15, 25, 35, 45 min, and 55 min) (Figure 1(c)). Findings revealed a significant increase in TPC from 8.17 to 16.19 mg GAE/g and in ABTS from 10.17 to 21.69 mg TE/g as the extraction time ranged from 5 to 35 min. However, there was no significant change when extraction time was extended from 35 to 45 min (*p* < 0.05). Simultaneously, both TPC and ABTS experienced a minor decline as the duration progressed from 45 to 55 min. The results indicated that with the prolonged ultrasound time, more phenolic compounds were decomposed [20, 24, 25]. Moreover, extended ultrasonic times enhance the dissolution of alcohol-soluble ingredients, thereby producing a large number of impurities and negatively affecting subsequent purification processes [25, 26]. Consequently, 35 min was selected as the optimal extraction time.

### 3.2. Model Fitting

In this study, RSM based on Boxbenhken design was used to optimize the UAE parameters on the recovery of TPC and ABTS. The independent variables comprised ultrasonic power (*X*_1_), extraction temperature (*X*_2_), and extraction time (*X*_3_). The experiment encompassed 17 runs, featuring three central points. The observed and predicted TPC and ABTS values spanned within the ranges of 6.50–16.56 mg GAE/g dm and 6.56–22.34 mg TE/g dm, correspondingly (Table 1).

**Figure 2:**
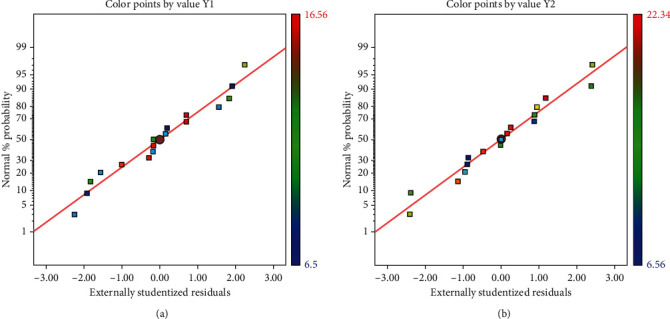
The normal probability plot of the studentized residuals for (a) total phenolic content (TPC) and (b) antioxidant capacity (ABTS radical scavenging activity) of the *R.A.* Poir extract.

The ANOVA results were used to check the adequacy of the suggested model (Table 2). The results revealed that a highly significant model was observed with an extremely low probability (*p* < 0.001). The high *R*^2^ (0.97-0.98) and adjusted *R*^2^ values (0.95-0.96), along with the minimal coefficient of variation (C.V. <10%) indicated the low deviation between the experimental and predicted values of the response [17, 26, 27]. Furthermore, the plotted points of the studentized residuals (Figures 2(a) and 2(b)) closely aligned with a straight line, indicating that the model was well fitted with the experimental results.

### 3.3. Effect of the Independent Variables on the TPC

**Figure 3:**
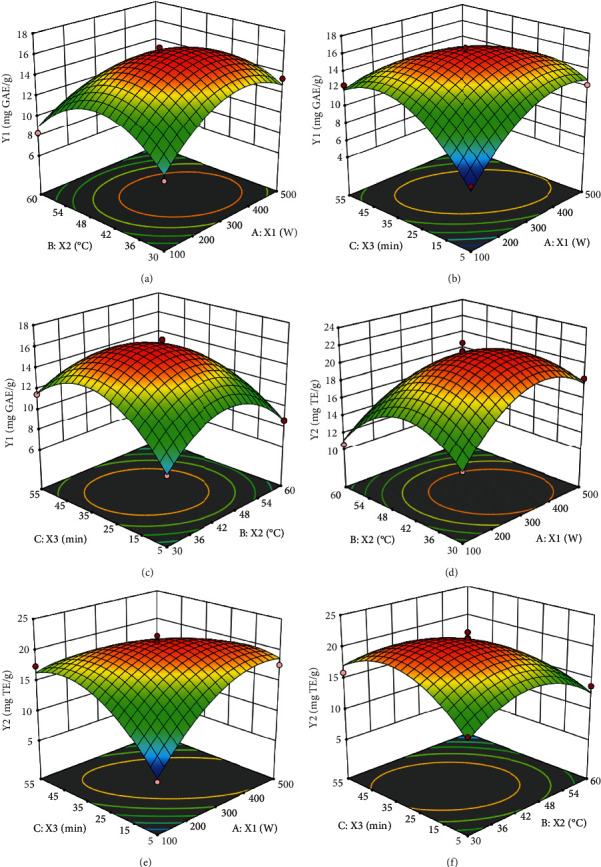
3D surface plot of relationships between ultrasonic parameters and TPC and ABTS: (a, d) given extraction time of 30 min; (b, e) given extraction temperature of 45°C; and (c, f) given ultrasonic power of 300 W.

Table 2 showcases the impact of independent variables (*X*_1_, *X*_2_, and *X*_3_) on the TPC. The linear term (*X*_1_) has a positive influence on the TPC, while cross terms (*X*_1_^∗^*X*_2_, *X*_1_^∗^*X*_3_, and *X*_2_^∗^*X*_3_) and the quadratic terms (*X*_1_^2^, *X*_2_^2^, and *X*_3_^2^) exert a substantial influence. The nonsignificant term (*X*_3_) was eliminated, and the second-order polynomial equation (Eq. (2)) was then generated. The relation between different factors and responses is elucidated by the three-dimensional (3D) response surface plots, as presented in Figure 3. As shown in Figure 3, the red color of the 3D surface indicated the highest value of the response value, and the blue color showed the lowest response value.

(2) $$\begin{array}{c} Y_{1}=16.09+0.733X_{1}-1.3X_{2}-2.86X_{1}^{2}-3.58X_{2}^{2}-3.76X_{3}^{2}-1.23X_{1}^{*}{\text{X}}_{2}-2.89X_{2}^{*}X_{3}-1.35X_{1}^{*}X_{3}. \end{array}$$

Figure 3(a) illustrates the marked interactive effect of ultrasonic power (*X*_1_) and extraction temperature (*X*_2_) on the TPC. Specifically, the TPC increased with increasing *X*_1_ (100-300W) at a fixed *X*_2_ (45°C). Similarly, the increase in *X*_2_ (30-45°C) at a fixed *X*_1_ (300W) also led to a gradual increase in the TPC and nearly reached a peak at the moderate *X*_2_ process (40-45°C). A further increase in *X*_1_ (300-500W) and *X*_2_ (45-60°C) resulted in a reduction in TPC. These might be caused by the acceleration of phenolic degradation with increasing extraction temperature and sonication power. Similar results were also reported for the extraction of phenolic compounds from bitter melon peel [28] and bee pollen [29].

Figure 3(b) shows the relationship between ultrasonic power (*X*_1_) and extraction time (*X*_3_), which affects the extraction efficiency and consequently TPC. As presented in Figure 3(b), the impact of *X*_1_ on the TPC is significant at longer *X*_3_, while the effect of *X*_1_ at shorter *X*_3_ is almost negligible. The extraction of phenolic compounds achieved the maximal value after 30 min of treatment and thereafter dropped. A similar trend was found in the UAE in *Epimedium brevicornum* maxim leaves [7] and watermelon rind [11]. A significant increase in TPC was obtained at high *X*_1_ (45°C) and increasing extraction time up to 30 min (Figure 3(c)). The extraction temperature of up to 45°C may increase and support the solubility and diffusion of phenolic compounds in the extraction solvent. However, an increased temperature of >60°C may destruct polyphenolic compounds, causing a decrease in antioxidant activity [30]. Furthermore, the sample cell membrane may break into small fragments under high extraction temperatures, which leads to increase impurities being extracted, thus a decrease in the recovery of phenolic compounds extracted. Similar results were reported for issoquercetin from *Ephedra alata* extracted with ultrasonics [26].

### 3.4. Effect of the Independent Variables on Antioxidant Activity

Figures 3(d)–3(f) describe the relationship between UAE parameters (ultrasonic power, temperature, and extraction time) on the radical scavenging activity (ABTS) of the extracts. As shown in Figure 2, the two linear terms (*X*_1_ and *X*_2_) have a positive influence on the ABTS. Meanwhile, all the quadratic terms (*X*_1_^2^, *X*_2_^2^, and *X*_3_^2^) and the cross-term coefficients (*X*_1_ × *X*_3_ and *X*_2_ × *X*_3_) have a negative influence on the radical-scavenging ABTS activity. The linear term (*X*_3_) and cross-term (*X*_1_ × *X*_2_) are not significant due to the value of *p* value greater than 0.05, and are similar to the results visualized in the elliptical contour plot.

(3) $$\begin{array}{c} Y_{2}=21.16+1.06X_{1}-2.70X_{2}-4.09X_{1}^{2}-4.31X_{2}^{2}-4.41X_{3}^{2}-4.90X_{2}^{*}X_{3}-2.13X_{1}^{*}X_{3}. \end{array}$$

As presented in the 3D plots for antioxidant activity (Figures 3(d)–3(f)), the extraction process variables affected the ABTS in a way similar to that in the case of the TPC. This may be due to the correlation between antioxidant activities and the phenolic compounds found in the leaf extracts of Rubus alceaefolius Poir [7, 11]. In Figure 3(d), the ABTS values increased with the increase of *X*_1_ and *X*_2_ and achieved the highest the antioxidant activity value at 323 W and 40.8°C. A further increase in *X*_1_ and *X*_2_ resulted in the reduction of the ABTS value, consistent with the RSM results of TPC (Figure 3(a)).

In Figure 3(e), it can be seen that the antioxidant activity is significantly enhanced with the increase in *X*_1_ (100-300W) at any *X*_3_ (*p* < 0.05). The ABTS value also increased with the increase of *X*_3_ (5-30min) at a fixed *X*_1_. However, higher *X*_1_ (400-500W) and extended *X*_3_ (40-55 min) resulted in low ABTS values due to the degradation of phenolics. This is similar to what was obtained for phenolic extraction from bitter melon [30] and *Celastrus hindsii* leaves [16].  Figure 3(f) illustrates the impact of *X*_2_ and *X*_3_ on the ABTS radical scavenging activity. At a fixed *X*_3_, the ABTS noticeably increased with the rise in *X*_2_ (30-40°C). When the highest ABTS was reached, any further increase in *X*_2_ led to a decrease in the ABTS. This might be due to the effect of high temperature on the nature and structure of the phenolic compounds [30]. The optimal *X*_2_ and *X*_3_ for the phenolic extraction were found to be at its central level.

### 3.5. Optimization and Comparison of UAE with Conventional Extraction

The optimal UAE conditions for maximum recovery of phenolic compounds and ABTS radical scavenging activity, as determined by the Design-Expert software, involved ultrasonic power (*X*_1_) of 320 W, temperature process (*X*_2_) of 40°C, and extraction time (*X*_3_) of 35.5 min. Under the optimal conditions, the experimental TPC and ABTS values were 16.68 mg GAE/g and 21.9 mg TE/g, respectively. These results closely matched the predicted values, demonstrating the models' accuracy with minimal prediction errors. Essentially, these response models can be effectively used to refine the UAE conditions for improved recovery of phenolic compounds and their antioxidant activity.

**Figure 4:**
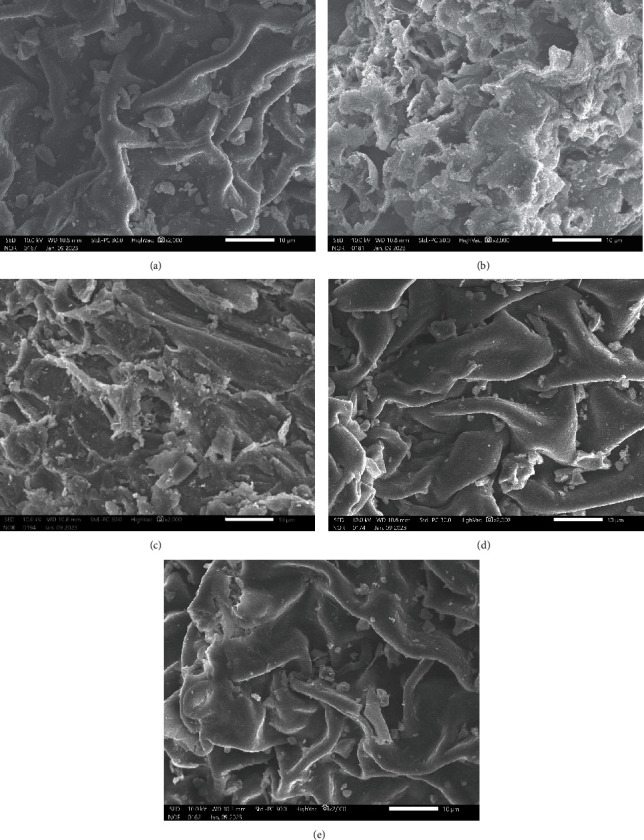
SEM images (×1,000) of (a) native, (b) at optimal UAE (320 W, 39.79°C, 35.32 min.), (c) extremely UAE (500 W, 60°C, and 55 min), (d) SE method, and (e) ME method.

The influence of the UAE method on the extraction yield of phenolic compounds and their antioxidant properties from *R.A.* Poir leaves were studied and compared with the conventional extraction methods (SE and ME). As shown in Table 3, the TPC and ABTS values using the UAE method are markedly elevated (37.35% and 33.15%, respectively) compared to the values achieved through SE and ME. The differences in TPC and ABTS values could be due to the variations in plant cell wall alterations observed through SEM. As presented in Figure 4(a), the cell surface of *R.A*. Poir leaf powder was smooth without cell wall deformation. In ME and SE, the plant cells have several holes, and a few cells were broken (Figures 4(c) and 4(d)). Meanwhile, the extracted leaf powder was seriously destroyed with small fragments at optimal extraction condition or extremely extraction condition (Figure 4(b)). The more ruptured plant cells resulted in a more efficient extraction. Our results also showed that the optimized UAE extracts presented the highest anti-*α*-amylase (99.10%) and *α*-amylase (0.90 mmol ACE/g) compared to the SE (76.34% and 0.45 mmol ACE/g) and ME (75.76% and 0.64 mmol ACE/g) extracts (*p* <0.05) (Table 3). According to Monteiro De Souza [28], *α*-amylase is related to the hydrolysis of low-molecular-weight carbohydrate products, such as glucose, maltose, and maltotriose units. The high levels of inhibition of *α*-amylase are involved in the low conversion of carbohydrates into glucose and delay their absorption in the intestine [26, 29]. Meanwhile, collagen contributes to skin elasticity and strength, and its degradation by collagenase is one of the main causes of intrinsic skin aging [26, 31]. As a result, the high *inhibition* of *collagenase* in the extract could lead to reducing skin damage or wrinkle formation. Therefore, the optimized UAE extract of *R.A.* Poir leaves might be more effective in reducing the degree of skin aging and blood glucose levels than the SE and ME extracts. Furthermore, the consumption of solvent and extraction time were significantly reduced by UAE in comparison with SE and ME. Therefore, *UAE* was a new alternative for the *extraction* of phenolic from *R.A.* Poir leaves.

### 3.6. GC-MS Analysis of Bioactive Compounds

The identification of bioactive compounds from the *R.A.* Poir leaf extracts obtained by optimal UAE conditions and conventional solvent extraction were analyzed with GC-MS. As presented in Table 4, it can be seen that 5 different phenolics and 3 flavonoids compounds were screened under different retention times (RT). Compared with the UAE method, the SE and ME had the same identified phenolic compounds, except for quinic acid and quercetin, which were not detected. In Table 4, the UAE method exhibited a higher individual phenolic content than the SE and ME methods. The most abundant compounds identified in the UAE extracts were gallocatechin (193.8 mg/100 g) and p-hydroxybenzoic acid (138.50 mg/100 g), followed by coumaroylquinic acid (129.7 mg/100 g) and quinic acid (120.9 mg/100 g), while the least abundant compound was vanillic acid (35.3 mg/100 g). In contrast, in the conventional solvent extraction methods (SE and ME), gallocatechin (131.78 mg/100 g; 190.45 mg/100 g) and p-hydroxybenzoic acid (108.03 mg/100 g; 113.54 mg/100 g) had the highest phenolic contents. The disparity was attributable to the degradation of bioactive compounds under long extraction time and high-temperature processes. Similar results were also reported for extracting bioactive compounds, especially phenolic compounds from *Psidium cattleianum* leaves using UAE and conventional aqueous–organic extraction [24]. Some previous studies showed that these phenolic compounds mentioned exhibited antimicrobial, antiosteoporotic, and control of human tumors [32–34]. Our findings also found benzaldehyde, 2-hydroxy-4-methyl, and 2,3-dihydro-benzofuran in the *R.A.* Poir leaf extract, which exhibited strong antioxidant capacity and anticancer [35, 36]. As can be concluded from the results, the identified compounds could be responsible for the ABTS radical scavenging activity of the extract obtained by the UAE method.

## 4. Conclusions

The response surface methodology was effectively utilized to optimize the extraction of phenolic compounds from *R.A.* Poir. The Box-Behnken design proved to be a highly beneficial tool for enhancing the parameters of ultrasonic-assisted extraction. The optimum conditions for UAE, as determined by RSM, included an ultrasound power of 320 W, an extraction temperature of 40°C, and a sonication duration of 35.5 min. Under these conditions, the experimental results agreed with the predicted values (*p* < 0.05). Additionally, the results indicated that the UAE method offered substantial advantages over SE and ME, particularly in terms of superior extraction efficacy and antioxidant activity of the extract in the shortest extraction time. Moreover, the leaf extracts were found to contain 5 phenolics and 3 flavonoid compounds. Hence, our research underlines the potential of the UAE procedure to enhance the extraction yield of phenolics and certain bioactive compounds with potent antioxidant capacities. Nevertheless, additional research is necessary for the full phytochemical characterization of *R.A.* Poir leaf extracts to confirm their possible applications.

## Figures and Tables

**Table 1:** Box–Behnken design of experimental conditions and observed responses for TPC and antioxidant capacity (ABTS radical scavenging activity) of *R.A.* Poir leaf extracted using ultrasonic-assisted extraction.

Run	Factors			Actual *Y*_1_ (mg GAE/g)	Predicted *Y*_1_ (mg GAE/g)	Prediction error (%)	Actual *Y*_2_ (mg TE/g)	Predicted *Y*_2_ (mg TE/g)	Prediction error (%)
	*X*_1_ (W)^a^	*X*_2_ (°C)^a^	*X*_3_ (min)^a^						
1	300 (0)	45 (0)	30 (0)	15.89 ± 0.61	16.08	1.18	20.65 ± 0.71	21.16	2.39
2	500 (+1)	45 (0)	55 (+1)	7.16 ± 0.31	7.15	-0.14	8.95 ± 031	8.97	0.22
3	300 (0)	45 (0)	30 (0)	16.56 ± 0.45	16.57	0.06	21.34 ± 0.59	21.39	0.23
4	300 (0)	45 (0)	30 (0)	15.45 ± 0.89	15.46	0.06	20.01 ± 0.35	20.59	2.87
5	100 (-1)	45 (0)	55 (+1)	12.50 ± 0.43	12.54	0.32	17.45 ± 0.31	17.48	0.17
6	300 (0)	30 (-1)	5 (-1)	8.50 ± 0.33	8.53	0.35	13.15 ± 0.34	13.19	0.30
7	300 (0)	60 (+1)	55 (+1)	6.60 ± 0.21	6.54	-0.92	6.80 ± 0.45	6.98	2.58
8	500 (+1)	45 (0)	5 (-1)	12.23 ± 0.25	12.45	1.77	17.67 ± 0.37	17.69	0.11
9	500 (+1)	60 (+1)	30 (0)	8.33 ± 0.30	8.35	0.24	10.11 ± 0.39	10.18	0.69
10	300 (0)	45 (0)	30 (0)	16.56 ± 0.37	16.55	-0.06	22.34 ± 0.67	22.33	-0.04
11	100 (-1)	30 (-1)	30 (0)	8.50 ± 0.21	8.53	0.35	13.05 ± 0.28	13.08	0.23
12	300 (0)	30 (-1)	55 (+1)	11.54 ± 0.24	11.98	3.67	15.99 ± 0.23	16.12	0.81
13	300 (0)	45 (0)	30 (0)	15.98 ± 0.39	15.99	0.06	21.45 ± 0.56	21.46	0.05
14	100 (-1)	45 (0)	5 (-1)	6.50 ± 0.21	6.46	-0.62	6.56 ± 0.15	6.51	-0.77
15	100 (-1)	60 (+1)	30 (0)	8.30 ± 0.23	8.31	0.12	10.55 ± 0.50	10.64	0.85
16	500 (+1)	30 (-1)	30 (0)	13.45 ± 0.20	13.40	-0.37	18.34 ± 0.78	18.37	0.16
17	300 (0)	60 (+1)	5 (-1)	8.65 ± 0.37	8.64	-0.12	13.80 ± 0.67	13.81	0.07

*^a^ X*_1_, *X*_2_, and *X*_3__,_ respectively, denote the ultrasonic power (W), extraction temperature (°C), and extraction time (min). The values are mean of three replications ± standard deviation.

**Table 2:** Results of the ANOVA for the response surface quadratic model.

Source	Total phenolic content (TPC)						Antioxidant activity (ABTS)					
	Parameter estimate	SS	DF	MS	*F* value	*p* value	Parameter estimate	SS	DF	MS	*F* value	*pvalue*
Model	16.10	221.72	9	24.635	48.27	<0.0001	21.16	445.05	9	49.45	35.03	<0.001
*X*_1_	0.733	3.605	1	3.605	7.06	0.032	1.06	8.95	1	8.95	6.34	0.04
*X*_2_	-1.30	12.777	1	12.78	25.04	0.001	-2.7	58.16	1	58.16	41.2	<0.001
*X*_3_	0.265^∗^	0.4608	1	0.4608	0.904	0.374^∗^	-0.25	0.495	1	0.495	0.35	0.573^∗^
*X*_1_^∗^ *X*_2_	-1.23	6.0516	1	6.0516	11.857	0.0108	-1.18	5.59	1	5.59	3.96	0.087^∗^
*X*_1_^∗^ *X*_3_	-2.89	30.636	1	30.637	60.028	<0.0001	-4.90	96.14	1	96.14	68.1	<0.001
*X*_2_^∗^ *X*_3_	-1.35	6.477	1	6.477	12.691	0.009	-2.13	18.23	1	18.23	12.92	0.0008
*X*_1_^2^	-2.86	33.817	1	33.818	66.261	<0.0001	-4.09	70.28	1	70.28	49.78	<0.0001
*X*_2_^2^	-3.58	54.842	1	54.842	107.456	<0.0001	-4.31	78.14	1	78.14	55.35	0.0001
*X*_3_^2^	-3.76	56.295	1	56.295	110.303	<0.0001	-4.41	82	1	82	58.09	0.0001
Residual		3.573	7	0.511				9.88	7	1.41		
LOF		2.669	3	0.8897	3.939	0.109^∗^		6.77	3	2.26	2.9	0.166^∗^
*R* ^2^		0.984						0.97				
Adj *R*^2^		0.964						0.95				
Pred *R*^2^		0.804						0.75				
C.V		5.87						7.85				

Df: degree of freedom; SS: sum of squares; MS: mean square; *R*^2^: coefficient of determination; Adj: adjusted *R*^2^; Predicted *R*^2^: Pred *R*^2^;*p* < 0.05 indicates statistical significance. ^∗^ stands for insignificant differences (*p* < 0.05).

**Table 3:** Comparison of the extraction efficiency of UAE with SE and ME methods.

Extraction method	TPC (mg GAE/g dm)	ABTS (mg TE/g dm)	Collagenase inhibition (%)	Alpha-amylase inhibition (mmol ACE/g)
UAE	16.68^a^ ± 1.12	21.90^a^ ± 1.01	99.10^a^ ± 2.14	0.90^a^ ± 0.07
SE	12.45^b^ ± 0.95	16.21^b^ ± 0.88	76.34^b^ ± 1.87	0.45^b^ ± 0.05
ME	10.45^c^ ± 1.00	14.64^c^ ± 0.96	75.64^b^ ± 1.96	0.64^b^ ± 0.04

Different letters in the same column indicate statistically significant differences between treatments (*p* < 0.05). The values are mean ± SD of duplicate runs.

**Table 4:** Chemical compounds of *R.A.* Poir leaf extract.

Peak	Compounds	Retention time (min)	Ultrasound-assisted extraction (UAE; mg/100 g)	Soxhlet extraction (SE; mg/100 g)	Maceration extraction (ME; mg/100 g)
1	Catechol	3.54	39.68 ± 1.03	39.45 ± 0.56	40.12 ± 1.03
2	2,3-Dihydro-benzofuran	4.95	36.3^a^ ± 0.78	28.31^b^ ± 0.41	35.21^a^ ± 0.56
3	p-Hydroxybenzoic acid	12.56	138.5^a^ ± 0.34	108.03^c^ ± 1.23	113.54^b^ ± 1.45
4	Gentisic acid	13.89	39.1^a^ ± 1.06	30.49^bc^ ± 0.41	32.45^b^ ± 0.56
5	Gallic acid	14.09	34.5^a^ ± 0.67	25.87^c^ ± 0.56	30.56^b^ ± 0.34
6	Vanillic acid	14.38	35.3 ± 0.65	34.56 ± 0.39	35.13 ± 0.67
7	Benzaldehyde, 2-hydroxy-4-methyl	14.94	88.9^a^ ± 2.01	62.23^b^ ± 1.01	56.98^c^ ± 1.15
8	Catechin	16.01	46.9^a^ ± 0.78	35.17^b^ ± 0.79	31.14^bc^ ± 0.94
9	Gallocatechin	17.31	193.8^a^ ± 2.14	131.78^b^ ± 2.11	190.45^a^ ± 1.21
10	Coumaroylquinic acid	18.32	129.7^a^ ± 1.31	14.49^b^ ± 0.33	13.90^b^ ± 0.76
11	Quinic acid	19.14	120.9^a^ ± 1.34	—	—
12	Quercetin	20.11	4.56^a^ ± 0.23	1.01^b^ ± 0.02	1.09^b^ ± 0.04

Different letters in the same row indicate statistically significant differences between treatments (*p* < 0.05). The values are mean ± SD of duplicate runs.

## Data Availability

The data supporting the findings of this study is included within the article.
